# Molecular mechanisms governing the differentiation and expansion of myeloid-derived suppressor cells

**DOI:** 10.3389/fcell.2025.1677201

**Published:** 2025-10-17

**Authors:** Ke Wang, Xiao Wang, Nan Sun

**Affiliations:** ^1^ Department of Cell Engineering, School of Life Sciences and Biotechnology, North Henan Medical University, Xinxiang, Henan, China; ^2^ Reproduction Medicine Center, Affiliated Hospital of Guangdong Medical University, Guangdong Medical University, Zhanjiang, Guangdong, China; ^3^ Department of Cell Engineering, North Henan Medical University, Xinxiang, Henan, China

**Keywords:** myeloid-derived suppressor cells, differentiation, expansion, tumor microenvironment, signal transduction, immunotherapy

## Abstract

Myeloid-derived suppressor cells (MDSCs) play critical roles in tumor immune evasion. These heterogeneous cells are broadly classified into granulocytic (G-MDSC), monocytic (M-MDSC), and their immature precursors, early-stage MDSCs (e-MDSCs). Elucidating their differentiation and expansion mechanisms is crucial for advancing cancer immunotherapy. This review examines the key signaling pathways (e.g., JAK/STAT, NF-κB, Notch), regulatory cytokines, metabolic factors, and epigenetic modifications central to MDSC biology. A comprehensive understanding of these intricate networks provides valuable insights into tumor immune evasion and facilitates the identification of novel therapeutic targets designed to overcome MDSC-mediated immunosuppression.

## 1 Introduction

MDSCs represent a pivotal component of the immune regulatory network, defined by their heterogeneity and potent capacity to suppress T cell activation and proliferation. These cells are broadly categorized into granulocytic (G-MDSCs) and monocytic (M-MDSCs) subpopulations based on morphological and phenotypic distinctions (Gabrilovich and Nagaraj, 2009; Youn et al., 2008). In various pathological settings—including cancer, chronic infections, and inflammatory disorders—both G-MDSCs and M-MDSCs deploy mechanisms involving reactive oxygen species, nitric oxide, and immunosuppressive cytokines to subvert immune responses, ultimately fostering immune dysfunction (Talmadge and Gabrilovich, 2013).

Mounting evidence underscores the role of critical signaling pathways—such as NF-κB, and JAK/STAT—in driving MDSC differentiation and expansion within the tumor microenvironment. Cytokines and growth factors, notably GM-CSF, IL-6, and VEGF, further orchestrate MDSC accumulation and enhance their suppressive functions, contributing to an immunosuppressive milieu that facilitates tumor progression (Gabrilovich et al., 2007; Condamine et al., 2015a). These findings highlight how intricate molecular networks converge to shape MDSC biology, thereby modulating anti-tumor immune responses and clinical outcomes.

Recent advances in single-cell sequencing technologies have shed light on the profound heterogeneity within MDSC populations, revealing distinct granulocytic and monocytic subsets with specialized immunomodulatory roles. However, knowledge gaps remain regarding the plasticity and lineage commitment of MDSCs, as well as the identification of robust biomarkers for different subsets. Elucidating these mechanisms and interactions—particularly in the context of other immune cell types—stands as a key challenge (Veglia et al., 2018). Accordingly, this mini-review provides a concise overview of the core molecular pathways governing MDSC differentiation and expansion, aiming to be accessible to a broad scientific and clinical audience. Addressing these gaps will not only expand our fundamental understanding of MDSC biology but also inform the design of targeted immunotherapies aiming to mitigate MDSC-driven immunosuppression in cancer and other diseases.

## 2 Biological characteristics of MDSCs

**FIGURE 1:**
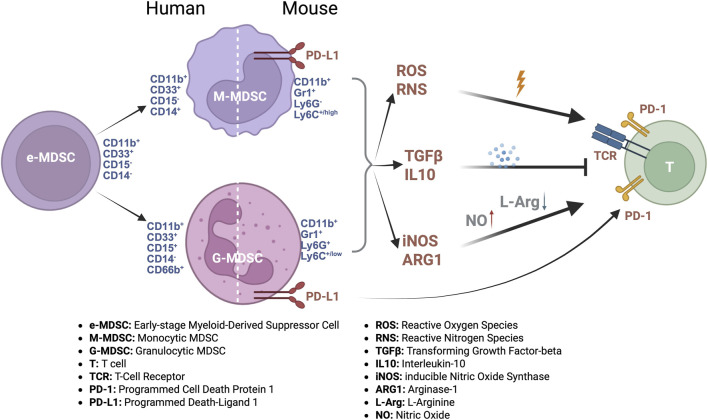
Phenotypic and functional characteristics of MDSC subsets. A schematic representation illustrating the heterogeneity and subsets of MDSCs in mice and humans. Mouse MDSCs are characterized primarily by the co-expression of CD11b and Gr-1, subdivided into granulocytic (G-MDSCs, CD11b+Ly6G + Ly6Clow) and monocytic (M-MDSCs, CD11b+Ly6G-Ly6Chigh) subsets. Human MDSCs broadly express CD11b and CD33 while displaying low HLA-DR expression. They are categorized into granulocytic (CD15^+^, CD66b+), monocytic (CD14^+^), and early-stage (CD14^−^CD15^−^) subsets. The early-stage subset is the precursor from which both granulocytic and monocytic MDSCs differentiate. Key functional immunosuppressive mechanisms utilized by each subset, such as ROS/RNS (Reactive Oxygen/Nitrogen Species) production, cytokine secretion (IL-10, TGF-β [Transforming Growth Factor-beta]), arginase-1, and iNOS (inducible Nitric Oxide Synthase) activities, are highlighted.

MDSCs are a heterogeneous population of immune cells distinguished by specific surface markers in mice and humans. In mice, MDSCs co-express CD11b and Gr-1 and are further classified into G-MDSCs (CD11b^+^Ly6G^+^Ly6C^+^/low) and M-MDSCs (CD11b^+^Ly6G^−^Ly6C^+^high) subsets, resembling neutrophils and monocytes, respectively (Youn et al., 2008). In humans, MDSCs are broadly identified by CD11b^+^CD33^+^HLA-DR^-^/low expression, and further subdivided into G-MDSCs (expressing CD15, CD66b), M-MDSCs (expressing CD14), and early-stage MDSCs (e-MDSCs; CD14^−^CD15^−^) (Movahedi et al., 2008; Damuzzo et al., 2014) (Figure 1), which are considered the immature common precursors to both M-MDSC and G-MDSC subsets. A key biological feature, particularly of G-MDSCs, is their low density, which distinguishes them from mature neutrophils of normal density and allows for their isolation from the peripheral blood mononuclear cell layer (Gabrilovich and Nagaraj, 2009; Sagiv et al., 2015). Moreover, in humans, the expression of lectin-type oxidized LDL receptor 1 (LOX-1) has been identified as an additional marker to help distinguish G-MDSCs from neutrophils (Condamine et al., 2016). Despite these classifications, overlapping marker expression and substantial heterogeneity necessitate the use of multiple markers and functional assays for precise identification.

Within the tumor microenvironment, MDSCs serve as key immunosuppressive regulators by inhibiting T cell and natural killer (NK) cell activity, and modulating antigen-presenting cells such as dendritic cells and macrophages (Solito et al., 2014; Kumar et al., 2016). They employ multiple mechanisms, including the production of reactive oxygen species (ROS) and reactive nitrogen species (RNS), which can modify T cell receptors and impair T cell function (Kumar et al., 2016). Moreover, MDSCs express high levels of arginase-1 (ARG-1) and inducible nitric oxide synthase (iNOS), depleting essential amino acids (e.g., arginine) and further suppressing T cell responses. These cells also secrete immunosuppressive cytokines, such as interleukin (IL)-10 and transforming growth factor-beta (TGF-β), which foster regulatory T cell (Treg) development and enhance their suppressive capacity (Ostrand-Rosenberg and Fenselau, 2018). Additionally, MDSCs upregulate immune checkpoint molecules like PD-L1, contributing to the exhaustion of effector T cells. Beyond direct immunosuppression, MDSCs support tumor progression by promoting angiogenesis, enhancing tumor cell survival, and facilitating metastasis, collectively creating a permissive niche for tumor growth and immune evasion (Alm et al., 2001) (Figure 1).

Outside of cancer, MDSCs play critical roles in chronic infections and autoimmune conditions. Their expansion in persistent infections can help prevent immunopathology by restraining hyperactive inflammatory responses; however, prolonged MDSC activity may hinder effective pathogen clearance. In autoimmune diseases, MDSCs can maintain immune tolerance by suppressing autoreactive T cells and modulating inflammatory processes—though dysregulated MDSC function can either exacerbate or ameliorate disease severity depending on the context (Gabrilovich and Nagaraj, 2009; Youn et al., 2008). Collectively, these diverse functions underscore MDSCs’ importance in shaping immune outcomes across pathological settings. A deeper understanding of MDSC biology, including their surface markers, functional mechanisms, and context-dependent effects, is essential for designing targeted therapies to harness their protective roles while mitigating detrimental immunosuppression, especially in the tumor microenvironment. It is important to note, however, that the immunosuppressive functions of MDSCs are not exclusively pathological. In physiological contexts such as pregnancy, MDSCs contribute to feto-maternal tolerance (Ostrand-Rosenberg et al., 2017). Furthermore, they can play protective roles by limiting excessive tissue damage in inflammatory conditions like graft-versus-host disease and certain autoimmune models (Highfill et al., 2010). Therefore, a comprehensive understanding of MDSC biology is essential for designing targeted therapies that can harness their protective roles while mitigating detrimental immunosuppression.

## 3 Mechanisms of MDSC differentiation

### 3.1 Origin of myeloid progenitor cells

MDSCs originate from common myeloid progenitor (CMP) cells in the bone marrow, which also give rise to various other myeloid lineages, including granulocytes, macrophages, and dendritic cells. Under steady-state conditions, CMPs differentiate into mature myeloid cells through tightly regulated processes involving specific transcription factors and signaling cues (Condamine et al., 2015b). However, in pathological states such as cancer, chronic infections, and inflammatory diseases, the differentiation pathway of CMPs is skewed towards the expansion of MDSCs (Talmadge and Gabrilovich, 2013). This aberrant differentiation is driven by a combination of intrinsic genetic programs and extrinsic environmental signals that disrupt normal myelopoiesis. The tumor microenvironment, rich in immunosuppressive factors and pro-inflammatory cytokines, plays a pivotal role in promoting the expansion and accumulation of MDSCs (Gabrilovich, 2017). These progenitor cells are diverted from their usual maturation pathways, leading to an increased production of MDSCs with potent immunosuppressive capabilities (Damuzzo et al., 2014; Li et al., 2021a). Understanding the origin and differentiation trajectory of MDSCs from CMPs is crucial for elucidating the mechanisms underlying their expansion in disease contexts and for identifying potential therapeutic targets to regulate their abundance and function.

### 3.2 Key signaling pathways and molecules

The differentiation of MDSCs is orchestrated by several key signaling pathways and molecular regulators, with the JAK/STAT, NF-κB, and Notch pathways playing central roles (Wang et al., 2019; Waight et al., 2013; Vasquez-Dunddel et al., 2013; Kujawski et al., 2008; Nefedova et al., 2004). The JAK/STAT pathway, particularly involving STAT3 and STAT5, is critical for promoting the expansion and immunosuppressive function of MDSCs. Activation of STAT3 by cytokines such as GM-CSF and IL-6 induces the expression of genes that drive MDSCs proliferation, survival, and suppressive activity (Vasquez-Dunddel et al., 2013). Similarly, STAT5 signaling contributes to the maintenance and expansion of MDSCs by regulating genes involved in cell survival and proliferation (Waight et al., 2013). The NF-κB pathway is another essential regulator, where its activation leads to the transcription of pro-inflammatory genes that support MDSC differentiation and function. NF-κB signaling enhances the production of immunosuppressive molecules like nitric oxide and ROS, which are pivotal for MDSC-mediated T cell suppression (Liu et al., 2023). Additionally, the Notch signaling pathway has been implicated in the regulation of MDSC differentiation. Notch receptors and their ligands are involved in cell fate decisions, and their activation promotes the expansion of MDSCs by influencing the expression of transcription factors that favor myeloid progenitor differentiation towards the MDSC lineage (Zhao et al., 2022a; Otani et al., 2022). Collectively, these signaling pathways interact in a complex network to regulate the differentiation, expansion, and functional programming of MDSCs, highlighting potential targets for therapeutic intervention to modulate their immunosuppressive effects in various diseases.

### 3.3 Cytokines and growth factors

Cytokines and growth factors are pivotal in shaping the differentiation and expansion of MDSCs within the tumor microenvironment and other pathological settings (Condamine et al., 2015b). GM-CSF, G-CSF, and M-CSF are key growth factors that influence myeloid progenitor cells to differentiate into MDSCs (Li et al., 2016). GM-CSF is essential for the expansion and survival of MDSCs by activating the JAK/STAT3 signaling pathway, which promotes the expression of genes involved in immunosuppression and cell proliferation (King et al., 2022). G-CSF contributes to the proliferation of G-MDSCs, while M-CSF is more involved in the differentiation of M-MDSCs (Abrams and Waight, 2012). In addition to these growth factors, pro-inflammatory cytokines such as IL-6, IL-1β, and vascular endothelial growth factor (VEGF) play significant roles in fostering an environment conducive to MDSC differentiation (Weber et al., 2021; Chen et al., 2019). IL-6 and IL-1β promote the activation of the STAT3 and NF-κB pathways, respectively, enhancing the immunosuppressive capabilities of MDSCs (Weber et al., 2021). VEGF not only supports angiogenesis but also contributes to the expansion of MDSCs by modulating the tumor microenvironment to favor their accumulation and function (Weber et al., 2021; Chen et al., 2019). These cytokines and growth factors create a feedback loop that sustains MDSC populations, thereby maintaining an immunosuppressive milieu that facilitates tumor growth and progression. Targeting these signaling molecules offers promising avenues for disrupting MDSC-mediated immunosuppression and improving the efficacy of immunotherapeutic strategies.

## 4 Mechanisms of MDSC expansion

### 4.1 Influence of the tumor microenvironment

**FIGURE 2:**
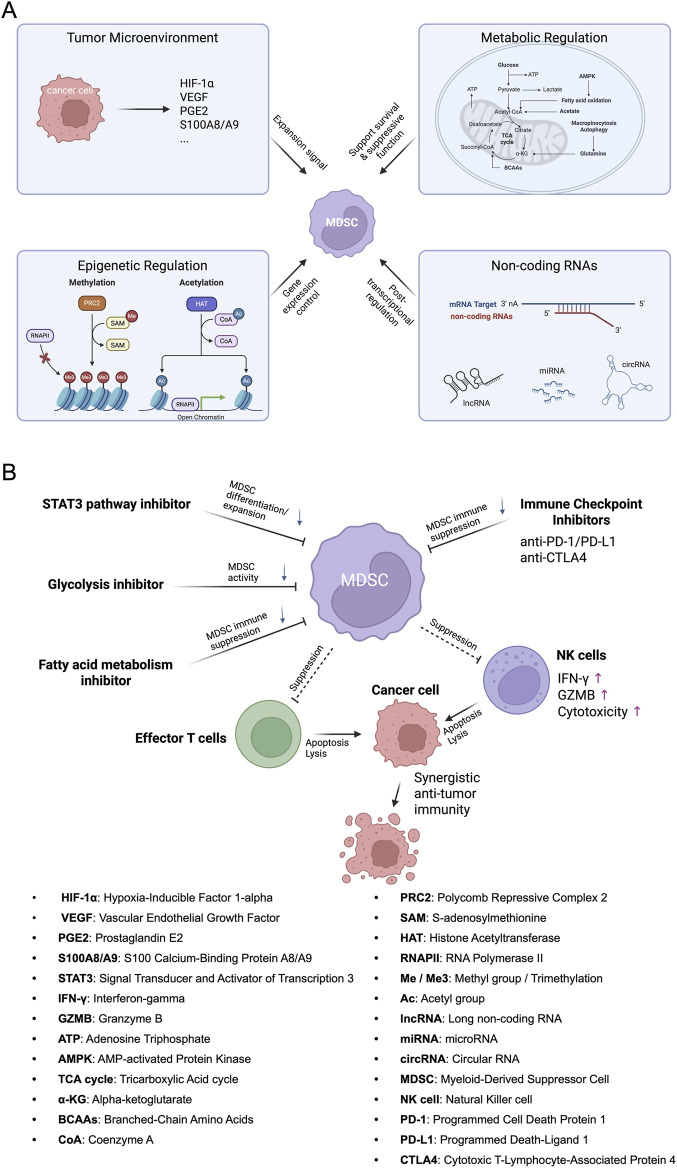
Molecular mechanisms regulating MDSC expansion and therapeutic strategies for enhanced anti-tumor immunity. **(A)** A detailed illustration summarizing key molecular and cellular processes responsible for MDSC expansion within the tumor microenvironment. This includes the influence of secreted factors (e.g., VEGF [Vascular Endothelial Growth Factor], S100A8/A9), hypoxia (HIF-1α [Hypoxia-Inducible Factor 1-alpha] activation), metabolic reprogramming pathways (glycolysis and lipid metabolism), epigenetic modifications (DNA methylation, histone acetylation/methylation), and regulation by non-coding RNAs (miRNAs [microRNAs], lncRNAs [long non-coding RNAs], circRNAs [circular RNAs]). Each process highlights its contribution to enhancing MDSC proliferation, survival, and suppressive function, emphasizing potential therapeutic targets for intervention. **(B)** Illustration showing combination approaches targeting MDSC differentiation and expansion pathways (STAT3 [Signal Transducer and Activator of Transcription 3], glycolysis, lipid metabolism inhibitors) alongside immune checkpoint inhibitors (anti-PD-1/PD-L1 [Programmed Cell Death Protein 1/Programmed Death-Ligand 1], anti-CTLA-4 [Cytotoxic T-Lymphocyte-Associated Protein 4]). These combined therapies act synergistically to reduce MDSC-mediated immunosuppression, enhancing effector T cell and NK cell functions, thus promoting robust anti-tumor immunity.

TME plays a pivotal role in the expansion of MDSCs through various secreted factors and hypoxic conditions (Netherby and Abrams, 2017). Tumor cell secretions, such as prostaglandin E2 (PGE2) and S100A8/A9 proteins, are critical in promoting MDSC expansion (Obermajer et al., 2012; Mao et al., 2014). PGE2, a lipid mediator, enhances the proliferation and suppressive functions of MDSCs by activating signaling pathways that upregulate immunosuppressive enzymes like ARG1 and inducible iNOS (Obermajer et al., 2012). Similarly, S100A8/A9 proteins, which are alarmins released by tumor cells, facilitate the recruitment and accumulation of MDSCs within the TME by binding to receptors such as RAGE and TLR4, thereby promoting their survival and expansion (Obermajer et al., 2012; Ostrand-Rosenberg and Sinha, 2009). Additionally, hypoxic conditions within the TME significantly contribute to MDSC expansion through the stabilization and activation of hypoxia-inducible factor 1-alpha (HIF-1α). HIF-1α induces the expression of genes that support MDSC proliferation, metabolism, and immunosuppressive activity under low oxygen levels (Zhang et al., 2025; Schwarz et al., 2023). The interplay between these secreted factors and hypoxia creates a conducive environment for the sustained expansion and functional enhancement of MDSCs, thereby facilitating tumor immune evasion and progression (Figure 2A).

### 4.2 Metabolic regulation

Metabolic reprogramming is a fundamental aspect of MDSC expansion and functionality, involving both glucose metabolism and lipid metabolism (Al-Khami et al., 2016). Glucose metabolism, particularly through the glycolytic pathway, is essential for MDSC survival and immunosuppressive functions. Enhanced glycolysis provides the necessary ATP and metabolic intermediates required for the rapid proliferation and activity of MDSCs. Additionally, glycolytic enzymes such as hexokinase and lactate dehydrogenase are upregulated in MDSCs, supporting their energy demands and enabling the production of immunosuppressive metabolites like lactate, which further inhibit T cell responses (Kim et al., 2024). On the other hand, lipid metabolism plays a crucial role in modulating the immunosuppressive capabilities of MDSCs. Accumulation of lipids within MDSCs leads to the activation of fatty acid oxidation pathways, which support their energy needs and enhance their suppressive functions (Bleve et al., 2025). Lipid droplets serve as reservoirs for bioactive lipids that can modulate signaling pathways involved in immune suppression. Moreover, altered lipid metabolism in MDSCs is associated with the upregulation of immunosuppressive molecules and the promotion of a pro-tumorigenic phenotype (Bleve et al., 2025). Together, the regulation of glucose and lipid metabolism orchestrates the expansion and functional adaptation of MDSCs, making metabolic pathways attractive targets for therapeutic intervention to mitigate MDSC-mediated immunosuppression (Figure 2A).

### 4.3 Epigenetic regulation

Epigenetic modifications, including DNA methylation and histone modifications, play a significant role in regulating the differentiation and expansion of MDSCs (Wang et al., 2024). DNA methylation, primarily occurring at CpG islands in gene promoter regions, modulates gene expression by silencing or activating specific genes involved in MDSC development and function (Smith et al., 2020). Hypermethylation of tumor suppressor genes or genes involved in differentiation pathways can promote the expansion of MDSCs by preventing their maturation into non-suppressive myeloid cells. Conversely, hypomethylation of genes associated with immunosuppressive functions, such as those encoding for ARG1 and iNOS, enhances the suppressive capacity of MDSCs (Smith et al., 2020). Histone modifications, including acetylation and methylation, further contribute to the epigenetic landscape that governs MDSC biology (Tian et al., 2023). Histone acetylation, mediated by histone acetyltransferases (HATs), generally promotes an open chromatin structure and active gene transcription, facilitating the expression of genes necessary for MDSC expansion and function (Tian et al., 2023). In contrast, histone methylation can either activate or repress gene expression depending on the specific residues modified. For instance, trimethylation of histone H3 lysine 4 (H3K4me3) is associated with active transcription, while trimethylation of histone H3 lysine 27 (H3K27me3) is linked to gene repression (Tian et al., 2023). These epigenetic modifications collectively regulate the transcriptional programs that drive MDSC differentiation, expansion, and immunosuppressive functions, highlighting the potential of epigenetic therapies in targeting MDSC-mediated immune suppression (Figure 2A).

### 4.4 Non-coding RNAs

Non-coding RNAs, including microRNAs (miRNAs), long non-coding RNAs (lncRNAs), and circular RNAs (circRNAs), are crucial regulators of MDSCs expansion and function (Shabgah et al., 2021). miRNAs modulate gene expression post-transcriptionally by binding to complementary sequences on target mRNAs, leading to their degradation or inhibition of translation. Specific miRNAs, such as miR-155 and miR-21, have been shown to enhance MDSC expansion by targeting negative regulators of the JAK/STAT and NF-κB signaling pathways, thereby promoting the transcription of genes involved in MDSC proliferation and immunosuppressive functions (Meng et al., 2020; Chen et al., 2015). lncRNAs influence MDSC biology through diverse mechanisms, including chromatin remodeling, transcriptional regulation, and acting as molecular sponges for miRNAs. For example, the lncRNA HOTAIR has been implicated in the stabilization of STAT3 signaling, which is essential for MDSC expansion and function (Thakuri et al., 2020). circRNAs contribute to MDSC regulation by serving as miRNA sponges or interacting with RNA-binding proteins to modulate gene expression. CircRNAs such as circ-0001273 have been identified to enhance MDSC-mediated immunosuppression by sponging miRNAs that normally inhibit MDSC-related gene expression (Li et al., 2021b; Li et al., 2020). These non-coding RNAs orchestrate intricate regulatory networks that control the differentiation, expansion, and suppressive activities of MDSCs (Figure 2A). Understanding the specific roles and mechanisms of non-coding RNAs in MDSC biology offers promising avenues for developing RNA-based therapeutics aimed at disrupting MDSC-mediated immune suppression in cancer and other diseases.

## 5 Endogenous and exogenous regulatory factors

MDSCs are regulated by a complex interplay of endogenous and exogenous factors that influence their expansion and function in various pathological contexts. Drug influences play a significant role in modulating MDSC populations. Certain chemotherapy agents, while primarily aimed at eliminating cancer cells, can inadvertently promote the expansion of MDSCs by inducing the release of growth factors and cytokines that favor myeloid progenitor survival and differentiation into MDSCs (Diaz-Montero et al., 2014). For instance, chemotherapeutic drugs such as gemcitabine and cyclophosphamide have been shown to increase MDSC levels, potentially contributing to immunosuppression and tumor recurrence post-treatment (Wang et al., 2017). Conversely, targeted therapies, including VEGF inhibitors, can directly impact MDSCs. VEGF inhibitors not only impair angiogenesis but also modulate the tumor microenvironment in a manner that reduces MDSC recruitment and function, thereby enhancing anti-tumor immune responses (Yang et al., 2025; Zhao et al., 2022b).

The microbiome constitutes another critical exogenous regulator of MDSCs. The gut microbiota interacts with the host immune system, influencing the differentiation and expansion of MDSCs through the production of microbial metabolites and modulation of systemic cytokine levels (Li et al., 2024). Certain bacterial species can enhance the immunosuppressive capacity of MDSCs by promoting the production of anti-inflammatory cytokines such as IL-10 and TGF-β, which are pivotal for MDSC-mediated immune regulation (Li et al., 2024). Additionally, dysbiosis, or an imbalance in the microbial community, has been associated with altered MDSC populations, highlighting the microbiome’s role in maintaining immune homeostasis and its potential as a therapeutic target to modulate MDSC activity.

Stress and inflammation are intrinsic endogenous factors that significantly contribute to the production and accumulation of MDSCs. Chronic inflammatory conditions create an environment rich in pro-inflammatory cytokines like IL-6, IL-1β, and TNF-α, which drive the expansion and activation of MDSCs (Nishida and Andoh, 2025). Persistent inflammation leads to the sustained activation of key signaling pathways, such as the JAK/STAT and NF-κB pathways, which are essential for MDSC proliferation and immunosuppressive function (Nishida and Andoh, 2025). Furthermore, stress-related hormones like glucocorticoids can modulate immune responses by enhancing MDSC survival and suppressive capabilities. The interplay between chronic inflammation and stress hormones creates a conducive milieu for MDSC accumulation, thereby facilitating immune evasion and exacerbating disease progression (Mundy-Bosse et al., 2011). Understanding these regulatory factors is crucial for developing strategies to mitigate MDSC-mediated immunosuppression and improve therapeutic outcomes in cancer and other chronic diseases.

## 6 Regulatory strategies for MDSCs

### 6.1 Interventions targeting differentiation and expansion

Targeting the differentiation and expansion of MDSCs presents a promising therapeutic approach to mitigate their immunosuppressive effects. Signal pathway inhibitors, such as STAT3 inhibitors, play a crucial role in this strategy. STAT3 is a key transcription factor that drives the proliferation, survival, and immunosuppressive functions of MDSCs (Vasquez-Dunddel et al., 2013). Inhibiting STAT3 disrupts these processes, leading to reduced MDSC accumulation and enhanced anti-tumor immune responses. Additionally, metabolic modulators that influence lipid and glucose metabolism are being explored to target MDSCs. Drugs that inhibit glycolysis or fatty acid oxidation can impair the energy metabolism of MDSCs, thereby diminishing their suppressive capabilities and viability (Li et al., 2021a; Bleve et al., 2025). For instance, glycolysis inhibitors like 2-deoxyglucose (2-DG) and fatty acid oxidation inhibitors such as etomoxir have shown potential in reducing MDSC-mediated immunosuppression (Kim et al., 2024). By interfering with the metabolic pathways essential for MDSC function, these interventions aim to limit the expansion and enhance the susceptibility of MDSCs to apoptosis, thereby restoring effective immune surveillance and response.

### 6.2 Combination immunotherapy strategies

Combining MDSC-targeting strategies with existing immunotherapies, particularly immune checkpoint inhibitors, holds significant potential for improving cancer treatment outcomes. Immune checkpoint inhibitors, such as anti-PD-1 and anti-CTLA-4 antibodies, work by reactivating exhausted T cells to mount effective anti-tumor responses (Nishida and Andoh, 2025). However, the presence of MDSCs in the tumor microenvironment can counteract the efficacy of these therapies by suppressing T cell activity. Integrating MDSC modulation with checkpoint blockade can synergistically enhance immune responses against tumors (Veglia et al., 2018; Gabrilovich, 2017). For example, combining STAT3 inhibitors with anti-PD-1 therapy has been shown to reduce MDSC levels and enhance T cell infiltration and activity within tumors (Bitsch et al., 2022). Similarly, using VEGF inhibitors alongside checkpoint inhibitors can decrease MDSC recruitment and function, thereby improving the overall immune-mediated tumor clearance (Saeed et al., 2021). These combination approaches aim to create a more favorable immune milieu by simultaneously removing immunosuppressive barriers and activating effector immune cells, thereby maximizing the therapeutic benefits and overcoming resistance mechanisms associated with monotherapies.

### 6.3 Future therapeutic directions

Looking ahead, the discovery and validation of novel molecular targets for regulating MDSCs represent critical areas for advancing therapeutic interventions. Emerging research is focused on identifying unique signaling molecules and regulatory networks that govern MDSC biology. Potential targets include novel transcription factors, surface receptors, and intracellular signaling proteins that are specifically involved in MDSC differentiation, expansion, and suppressive functions (Ozbay Kurt et al., 2023). Additionally, the role of epigenetic modifiers and non-coding RNAs in MDSC regulation is gaining attention, offering new avenues for intervention (Wang et al., 2024; Meng et al., 2020; Thakuri et al., 2020; Li et al., 2020). High-throughput screening and advanced genomic techniques are facilitating the identification of these targets, enabling the development of highly specific and effective MDSC inhibitors. Furthermore, personalized medicine approaches that consider the unique immune landscape of individual patients could enhance the precision and efficacy of MDSC-targeted therapies (Veglia et al., 2018). Validating these novel targets through preclinical and clinical studies is essential to translate these findings into effective treatments (Figure 2B). Ultimately, expanding our understanding of the intricate molecular mechanisms underlying MDSC regulation will pave the way for innovative therapies that can effectively disrupt MDSC-mediated immunosuppression and improve patient outcomes in cancer and other diseases.

## 7 Discussion

In summary, the differentiation and expansion of MDSCs are governed by a complex network of molecular mechanisms, including key signaling pathways such as JAK/STAT3, NF-κB, and Notch, as well as various cytokines and growth factors like GM-CSF, IL-6, and VEGF. These pathways and factors collectively orchestrate the skewed myelopoiesis that leads to the accumulation of immunosuppressive MDSCs within the tumor microenvironment and other pathological settings. However, current research faces several limitations, including the reliance on murine models that may not fully recapitulate human MDSC biology, the heterogeneity of MDSC populations which complicates their identification and characterization, and significant challenges in translating preclinical findings into effective clinical therapies. Additionally, the dynamic and context-dependent nature of MDSC regulation remains poorly understood, hindering the development of targeted interventions. Looking forward, future studies should aim to elucidate the precise molecular drivers of MDSC plasticity and differentiation in diverse disease contexts, identify reliable biomarkers for distinct MDSC subsets, and explore novel therapeutic targets that can effectively disrupt MDSC-mediated immunosuppression. Advances in single-cell genomics and proteomics, along with the development of more representative human models, will be critical in addressing these gaps. Ultimately, advancing our understanding of MDSC biology and their complex interplay with the tumor microenvironment will drive the innovation for innovative strategies to enhance immunotherapeutic efficacy and improve outcomes for patients with cancer and other immune-related diseases.
